# 

**Published:** 1895-04

**Authors:** 


### American Medical Association Meeting, at Baltimore, May 7th to 10th, 1895.

The coming meeting of the American Medical Association, to be held in Baltimore, in May, will undoubtedly be very largely attended and promises to be one of the most successful and interesting meetings of recent years. A great many were prevented from attending the San Francisco meeting owing to the length of the journey, but this will not be the case with Baltimore, as the running time is only about twenty-nine hours from St. Louis.

A great many interesting papers will be read and a general good time is already assured.

After adjournment cheap round trip tickets can be procured from Baltimore to New York and return, and members will be accorded the privilege of a stop-over at Washington and will undoubtedly be given the privilege of taking a steamer at Washington and going down the Potomac river to Old Point Comfort, on the sea-shore, and home via Richmond, Charlottesville, White Sulphur Springs and Cincinnati.

Already the Big Four route and Chesapeake & Ohio Ry. officials are figuring on rates and arrangements, and it is their intention to fix up an attractive itinerary.

The scenery along the Chesapeake & Ohio Ry. is beautiful at any season of the year, but it is particularly attractive in May, especially in Virginia. All vestibuled throughout, lighted by electricity, carry dining cars, elegant sleeping cars and observation cars, and the entire line is operated on the Block Signal System. The Chesapeake & Ohio is essentially a tourist route and is famous for the magnificent scenery through the Blue Ridge and Alleghany Mountains and the many noted health and pleasure resorts and famous battlefields scattered along the line.

The Big Four route is the western connection of the Chesapeake & Ohio, and the combined lines present the most attractive and comfortable route to the Baltimore meeting.

For further particulars, maps, etc., address

THE. C. WELLS,
Traveling Passenger Agent C. &. O. Ry.,
Dallas, Texas.

